# Lineage Differentiation and Genomic Vulnerability in a Relict Tree From Subtropical Forests

**DOI:** 10.1111/eva.70033

**Published:** 2024-11-01

**Authors:** Xian‐Liang Zhu, Jing Wang, Hong‐Feng Chen, Ming Kang

**Affiliations:** ^1^ Key Laboratory of National Forestry and Grassland Administration on Plant Conservation and Utilization in Southern China Guangzhou China; ^2^ University of Chinese Academy of Sciences Beijing China; ^3^ South China National Botanical Garden Guangzhou China; ^4^ State Key Laboratory of Plant Diversity and Specialty Crops, South China Botanical Garden Chinese Academy of Sciences Guangzhou China

**Keywords:** *Bretschneidera sinensis*, demographic history, genomic divergence, genomic offset, mutation load, relict tree

## Abstract

The subtropical forests of East Asia are renowned for their high plant diversity, particularly the abundance of ancient relict species. However, both the evolutionary history of these relict species and their capacity for resilience in the face of impending climatic changes remain unclear. Using whole‐genome resequencing data, we investigated the lineage differentiation and demographic history of the relict and endangered tree, *Bretschneidera sinensis* (Akaniaceae). We employed a combination of population genomic and landscape genomic approaches to evaluate variation in mutation load and genomic offset, aiming to predict how different populations may respond to climate change. Our analysis revealed a profound genomic divergence between the East and West lineages, likely as the result of recurrent bottlenecks due to climatic fluctuations during the glacial period. Furthermore, we identified several genes potentially linked to growth characteristics and hypoxia response that had been subjected to positive selection during the lineage differentiation. Our assessment of genomic vulnerability uncovered a significantly higher mutation load and genomic offset in the edge populations of *B. sinensis* compared to their core counterparts. This implies that the edge populations are likely to experience the most significant impact from the predicted climate conditions. Overall, our research sheds light on the historical lineage differentiation and contemporary genomic vulnerability of *B. sinensis*. Broadening our understanding of the speciation history and future resilience of relict and endangered species such as *B. sinensis*, is crucial in developing effective conservation strategies in anticipation of future climatic changes.

## 1 Introduction

The subtropical forests of East Asia, distinguished by their high plant diversity and endemism, are home to abundant ancient relict species, notably including *Ginkgo biloba*, *Metasequoia glyptostroboides*, and *Glyptostrobus pensilis* (Lu et al. 2018; Qian et al. 2019). These relict species may have survived through the Quaternary climatic changes, partly due to the absence of ice sheets in the region during the glacial periods (Clark and Mix 2002; Ehlers and Gibbard 2007). They are commonly known as “living fossils” because their morphological characteristics have remained virtually unchanged over long periods of time. In recent decades, the evolutionary origin and historical demography of relict plants in East Asia have attracted much attention (Qiu et al. 2017; Tang et al. 2018). A number of population genetic and phylogeographic studies have shown that the complex geological history and environmental heterogeneity of the subtropical region of East Asia may have played an important role in the lineage diversification in relict species (Qiu et al. 2017; Cao et al. 2020; Yin et al. 2021; Feng et al. 2024). However, the evolutionary origins and demographic histories of relict forest species in East Asia are still not well understood.

Many relict species were widespread during the last glacial period but now are often characterized by narrow geographic distribution, small population sizes, and limited dispersal capacity. This makes them high‐priority targets for biodiversity conservation. Therefore, a comprehensive assessment of genomic load is crucial for their effective conservation efforts. Recent advances in sequencing technologies and bioinformatic tools have greatly improved our ability to understand the fate of endangered populations and to inform conservation management from a genomic perspective, leading to the emergence of the rapidly expanding field of conservation genomics (Primmer 2009; Allendorf, Hohenlohe, and Luikart 2010; Ouborg et al. 2010; Supple and Shapiro 2018; Hohenlohe, Funk, and Rajora 2021). Alongside high‐quality reference genomes, whole‐genome resequencing data have enabled accurate measurement of genomic metrics for conservation. These metrics include runs of homozygosity (ROH; Curik, Ferenčaković, and Sölkner 2014), mutation load (Gaut, Díez, and Morrell 2015), evolutionary constraint (Huber, Kim, and Lohmueller 2020), and nucleotide diversity (*π*). Of these, the combination of ROH, which reflects the true inbreeding coefficient (*F*_ROH_), and mutation load, which correlates with fitness, is considered the most promising way to develop conservation genomics in endangered species (Wootton et al. 2023). Small and isolated populations are predicted to accumulate mutation load due to increased genetic drift (Lynch, Conery, and Burger 1995). However, as levels of inbreeding are also predicted to increase in small populations, strongly deleterious mutations could become exposed to purging, leading to a reduction in mutation load and potential mitigation of the harmful effects of inbreeding depression (Grossen et al. 2020). On the other hand, deleterious mutations can become fixed due to genetic drift in small populations (Lynch, Conery, and Burger 1995). Mutation load can be divided into the reduction in expected fitness due to expressed deleterious alleles (realized load or expressed load) and the recessive deleterious effects hidden in heterozygosis (masked load or potential load) (Bertorelle et al. 2022). However, if levels of inbreeding increase in small populations, much of the previously hidden or masked load can become realized over time, which may result in inbreeding depression (Mathur and DeWoody 2021; Bertorelle et al. 2022). It is therefore useful to differentiate between realized load and masked load when trying to understand the genomic health of endangered species.

To ensure comprehensive genomic diversity, it is important to also consider the adaptive potential of relict species in the face of anticipated future climate changes. Genomic offset (also known as genetic offset or genomic vulnerability) quantifies the disruption of genotype–environment associations under environmental change, and can serve as a predictive metric for maladaptation to future climatic conditions (Fitzpatrick and Keller 2015; Rellstab, Dauphin, and Exposito‐Alonso 2021; Gallegos, Hodgins, and Monro 2023). The prediction is based on the assumption that the population has optimally adapted to its current environment, without considering phenotypic plasticity or directly measuring individual fitness (Hoffmann, Weeks, and Sgrò 2021; Rellstab, Dauphin, and Exposito‐Alonso 2021; Láruson et al. 2022). This implies that any changes in the composition of allele frequency will result in reduced fitness (Capblancq et al. 2020). Genomic offset may be particularly useful for non‐model, protected, or long‐lived species where experimentation is impractical or even impossible. It could be used to, for example, identify threatened populations, select source populations for assisted migration/gene flow or recolonization, and identify suitable habitats in a future and changing environment. In recent years, a growing number of studies have used population‐level genomic data to evaluate genomic offset to climate change for forest tree species (Borrell et al. 2020; Pina‐Martins et al. 2019; Ingvarsson and Bernhardsson 2020; Jia et al. 2020; Dauphin et al. 2021; Gougherty, Keller, and Fitzpatrick 2021; Varas‐Myrik et al. 2022; Guo et al. 2023; Hung et al. 2023; Yuan et al. 2023). These studies not only provide important insights into how organisms adapt to their environments at the genomic level but also help inform and enhance conservation practices in anticipation of future environmental changes. However, such studies have been rarely conducted in relict tree species.

*Bretschneidera sinensis*, a tree species belonging to the Akaniaceae family, is an ancient Tertiary relict that is endemic to East Asia and is a key protected wild plant in China (Wang et al. 2018). Like many relict species, populations of this species in the wild are typically small and isolated, which presents challenges for natural regeneration (Qiao et al. 2009). Nevertheless, this species has a relatively wide distribution and mainly inhabits evergreen forests on valley or streamside slopes, at elevations ranging from 500 to 2000 m, south of the Yangtze River Basin in China, with minor occurrences in northern Vietnam and Myanmar (Gong et al. 2015; Guo et al. 2020). After being introduced to Britain by plant hunters in the mid‐19th century, *B. sinensis* has been progressively domesticated in Europe as a common landscape tree, demonstrating its potential for adaptation to diverse climates. This makes *B. sinensis* an exemplary model for studying widespread relict species, facilitating research into lineage differentiation patterns, genomic load, and adaptation to past and future climatic conditions influenced by environmental heterogeneity. In this study, we aim to address the following questions: (1) How differentiated are the different lineages of *B. sinensis*, and do these lineages have distinct demographic histories? (2) What characterizes the current genomic diversity of the populations? We are particularly interested in genomic inbreeding and mutation load. (3) How will *B. sinensis* be affected by future climate changes in terms of genomic offset? By investigating these factors, we can gain a better understanding of the evolutionary potential of *B. sinensis*, and improve our capacity to guide conservation efforts for this relict and endangered species.

## 2 Materials and Methods

### 2.1 Sample Information and Genomic Data Processing

Whole‐genome resequencing data of 154 *B. sinensis* individuals from 13 populations in China were obtained from Liu et al. (2022). Adapters and low‐quality sequences were removed from the raw data using Trimmomatic v0.39 (Bolger, Lohse, and Usadel 2014). The clean reads were then aligned to the *B. sinensis* reference genome (GenBank: GCA_018105755.1) using BWA v0.7.17 (Li and Durbin 2009). After removing duplicate alignments with Picard v.2.18.11 (http://broadinstitute.github.io/picard/) and sorting with SAMtools v1.9 (Li et al. 2009), the *HaplotypeCaller* module of GATK v4.2.6 (McKenna et al. 2010) was used to call single nucleotide polymorphisms (SNPs). The raw SNPs were filtered using GATK's *VariantFiltration* module with the following parameters: “QD < 2.0 || FS > 60.0 || MQ < 40.0 || MQRankSum < −12.5 || ReadPosRankSum < −8.0”, resulting in 1,116,047,066 SNPs. We used VCFtools v0.1.13 (Danecek et al. 2011) to remove loci with depths of < 5 and > 100, quality < 30, and missing rate > 20%. This resulted in 48,070,027 high‐quality biallelic SNPs. For subsequent analyses, we further used VCFtools and Plink v1.9 (Chang et al. 2015) to filter by minor allele frequency, degree of linkage disequilibrium between loci, and missing rate to obtain different SNP sets (Table S1).

### 2.2 Population Genetic Analysis

Population structure analysis was conducted using Admixture v1.3.0 (Alexander, Novembre, and Lange 2009), with the presumed number of substructures (*K*) set from 1 to 10. We also performed principal component analysis (PCA) without a priori assumptions using GCTA v1.94 (Yang et al. 2011). Nucleotide diversity (π), indicative of genetic diversity, was quantified for each population using PIXY v1.2.7 (Korunes and Samuk 2021). A further inquiry into genetic variation and differentiation between lineages involved the use of pure individuals; these were those with a lineage composition > 0.95 in the Admixture (Figure S1), thus minimizing the potential influence of recent gene flow events. To avoid sample bias in estimation, 40 pure individuals were selected each from the East and West lineages. Subsequent calculation of π for both the East and West lineages, as well as the relative genetic differentiation (*F*_ST_) and absolute divergence (*D*_XY_) between the lineages, were performed using PIXY. Natural selection within a population genomic framework was detected using CEGA v1.2 (Zhao, Chi, and Chen 2023), a novel methodology that evaluates the role of natural selection based on the maximum likelihood method with Hudson‐Kreitman‐Aguad‐type composite likelihood ratio tests. In the CEGA analysis, the designation “‐i1” was assigned to represent the East lineage, while “‐i2” was used to denote the West lineage for comparison. The selected regions with Normalized lambda (Nλ) values < −2.3263 (positive selection) or > 2.3263 (balancing selection) at 99% confidence were identified following Zhao, Chi, and Chen (2023). Since the removal of neutral variants linked to deleterious alleles in regions with low recombination rates could also cause low λ, the regions of positive selection identified by CEGA may have been partially affected by background selection. Additionally, Tajima's *D* (Fumio 1989) values were computed using VCFtools, with positive and negative values indicative of balancing selection and directional selection, respectively. The calculations of π, *F*_ST_, *D*_XY_, Nλ, and Tajima's *D* values were performed using non‐overlapping 100‐Kb windows. Finally, for functional enrichment analysis of selected genes, GO and KEGG tools incorporated in TBtools v2.026 (Chen et al. 2020) were utilized.

### 2.3 Gene Flow

Treemix v1.13 (Pickrell and Pritchard 2012) was used to infer migration events between populations. Ten iterations were run for each of the assumed 0–10 migration events (*m*), with resampling blocks of 500 SNPs per iteration and “‐global ‐se ‐noss” to calculate standard errors (SE) and avoid overcorrection for sample size. Because of the absence of outgroups, we did not assign a root to the maximum likelihood tree. Then, ∆*m* was calculated using the R package OptM v0.1.6 (Fitak 2021) to estimate the optimal number of migration edges. Meanwhile, we also used Dsuite v0.3 (Malinsky, Matschiner, and Svardal 2021) to perform the ABBA‐BABA test between populations. The *D*‐statistic was estimated using the *Dquartets* module, which heuristically explores potential gene introgression between all “(((P1, P2) P3) P4)” combinations without outgroups. To minimize false positives, only cases with *p* < 0.001 were considered as significant gene flow events.

### 2.4 Demographic History

To infer the demographic history of different *B. sinensis* lineages, we randomly selected 15 individuals each from the East and West lineages and used three complementary methods for analysis. Firstly, we utilized SNeP v1.11 (Barbato et al. 2015), an algorithm‐based linkage disequilibrium, to infer the effective population size (*N*_e_) over the last 1000 generations. We employed Plink to convert the locus data into “ped” and “map” formats before running SNeP with its default parameters. Secondly, we made use of SMC++ v1.15.4 (Terhorst, Kamm, and Song 2017). This method is based on coalescent hidden Markov models. Given that some mask regions in the genome could potentially influence the process of demographic history inference, we utilized the SNPable tool (https://lh3lh3.users.sourceforge.net/snpable.shtml) in conjunction with the *aln* and *samse* tools of the BWA software to identify the mask within the *B. sinensis* genome. In SMC++, we employed the *vcf2smc* command to convert the locus data into the input format required by SMC++ and specified genomic mask information via the *‐m* parameter, subsequently using the *estimate* command to evaluate the demographic histories of both lineages independently. Furthermore, the divergence time of both lineages was estimated using the *split* command. Our third method was PSMC v0.6.5 (Li and Durbin 2011), an approach for inferring ancient *N*_e_ fluctuations by calculating the time since the most recent common ancestor of alleles. We ran the parameters: ‐N25 ‐t15 ‐r5 ‐p “4 + 25 × 2 + 4 + 6”. Given the tool's limitation where only one individual could be analyzed at a time, we ran 10 replicates of PSMC for each of the 15 individuals to ensure accurate *N*_e_ estimates. For both SMC++ and PSMC, we adopted an assumed mutation rate of 1.39e‐9 per site per generation and a generation duration of 20 years specific for *B. sinensis* (Liu et al. 2022). Furthermore, we randomly sampled six individuals from each population and then estimated the most recent *N*_e_ for each of the 13 populations using SNeP.

### 2.5 Genomic Inbreeding

ROH refers to the genomic region where all loci are homozygous. We utilized Plink's *homozygosity* parameter to identify ROHs for all individuals. To pinpoint ROHs exceeding 10 Kb in length, we calculated a 50‐SNP window, excluding heterozygous loci. We calculated ROHs spanning from 10 to 100 Kb, 100 to 200 Kb, and those > 200 Kb as short, medium, and long ROHs, respectively. Typically, long ROHs reflect recent inbreeding, whereas short ROHs indicate ancient inbreeding (Meyermans et al. 2020). Using RectChr v1.36 (https://github.com/BGI‐shenzhen/RectChr), we visualized the distribution of ROHs across the genome. The level of genome‐wide inbreeding was quantified as *F*_ROH_, which was calculated by dividing the cumulative length of all ROHs (> 10 Kb) within an individual by the total genome length. In addition, we computed another classical individual‐based inbreeding coefficient (*F*_IS_) using Plink's *het* parameter. Higher *F*_IS_ values indicate reduced heterozygosity among individuals.

### 2.6 Mutation Load

Mutation load is the decrement in the fitness of individuals because of harmful mutations that interfere with gene function. To identify these deleterious mutations, we utilized allele genotypes that were homozygous in over 50% (> 77) of individuals to represent the ancestral state. Considering that a certain number of harmful mutations may exist in the reference genome—thereby causing detection bias—loci were excluded when their ancestral state did not align with the reference genome. We constructed a database based on the annotation of *B. sinensis* genomes using SNPEff v5.1 (Cingolani et al. 2012), then annotated the SNPs in each individual. Variants annotated as synonymous were defined as benign synonymous (SYN) mutations. For missense variants, we used Hu et al. (2021) script to assess the Grantham Score (GS). GS is a method used to evaluate the impact of a mutation based on the physical or chemical similarity between amino acids. A higher GS for mutations signifies higher deleteriousness (Grantham 1974). We classified a GS between 5 and 150 as slightly deleterious tolerated (TOL) mutations and a GS greater than 150 as deleterious (DEL) mutations. Those that resulted in a stop gain, splice acceptor variant, or splice donor variant were directly classified as severely deleterious loss‐of‐function (LOF) mutations. Simultaneously, we sorted mutations into heterozygous and homozygous categories based on the derived genotype. We estimated the mutation loads of TOL, DEL, and LOF according to the ratio of derived homozygous loci to total derived loci (two per homozygous locus and one per heterozygous locus), following the method described by Feng et al. (2019). By comparing the proportion of deleterious heterozygous and homozygous mutations to all SYN, respectively, we calculated masked loads and realized loads.

### 2.7 Genomic Offset

To assess the genomic offset (i.e., genomic vulnerability) of *B. sinensis* populations to future climate conditions, we utilized a gradient forest (GF) machine learning approach. This method allowed us to predict the genomic offset of locally adapted populations under future climate scenarios by capturing the nonlinear relationship between current climatic variables and allele frequencies (Láruson et al. 2022). We first calculated allele frequencies for all populations using a dataset of 82,878 SNPs that did not contain any missing loci and was heavily linkage disequilibrium pruned (Table S1). The GF model was then constructed using the R package gradientForest v.0.1.37 (Ellis, Smith, and Pitcher 2012). This was involved building a 500 replicate regression tree between current climatic variables and allele frequencies, setting the *corr.threshold* at 0.7. Redundant variables (|*R*| > 0.7 and low for importance) were removed by calculating the Spearman's correlation (*R*) between the climatic variables. To explore the maladaptive estimates of genomic variation in response to future climate, we performed further GF simulations using both adaptive and neutral loci. Adaptive loci were identified using the redundancy analysis (RDA) model in R package vegan v2.5‐6 (Oksanen, Blanchet, and Friendly 2019). In the RDA model, the first three axes of representative climate variables were used to detect adaptive SNPs, and their significance was tested through 999 permutations. Neutral loci, in contrast, were randomly selected from SNPs annotated as “intergenic regions” by SNPEff. Climate variables for both the recent current period (1970–2000) and anticipated future period (2081–2100, BCC‐CSM2‐MR model) periods were downloaded from WorldClim v2.1 with a resolution of 2.5 min (Fick and Hijmans 2017). Euclidean distances between current and future scenarios of allelic turnover were measured as genomic offsets, with a grid (~4.6 × 4.6 km) as the unit across the entire distribution of *B. sinensis*. We averaged the genomic offset values derived from the four shared socio‐economic pathways (SSPs). A circular distribution with a 20 km radius was then plotted for each population in ArcGIS v10.7 (Esri, USA). Consequently, the mean genomic offset for each population was determined (Aguirre‐Liguori, Ramírez‐Barahona, and Gaut 2021). Genomic offset depends on the genetic composition of populations and the magnitude of the expected environmental shifts. Therefore, populations with higher genomic offsets are expected to be more vulnerable to climate change (Fitzpatrick and Keller 2015).

## 3 Results

### 3.1 Genomic Landscapes of Diversity and Differentiation

**FIGURE 1:**
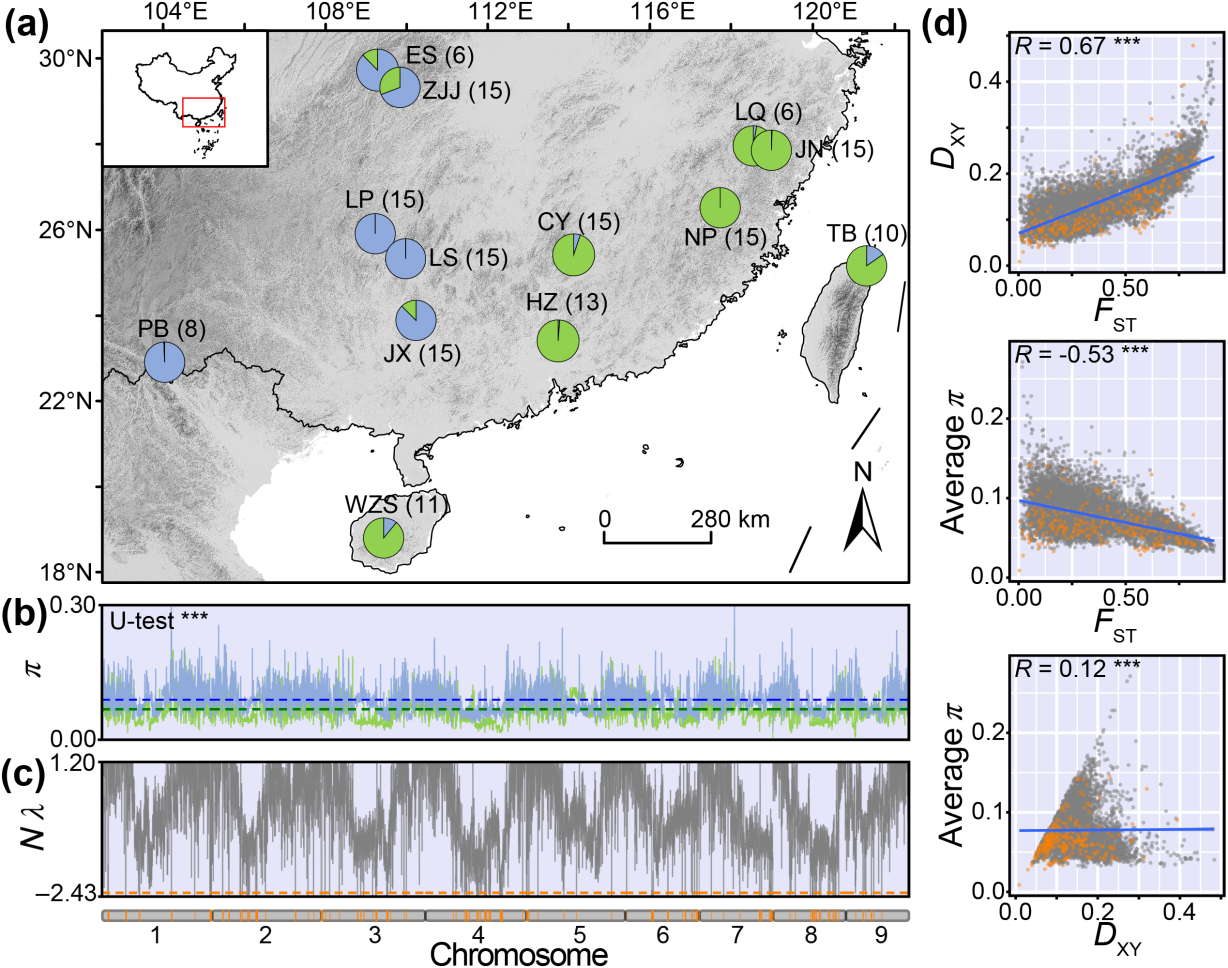
Geographic distribution and genomic variation patterns in *B. sinensis*. (a) Location of the 13 sampled populations. Numbers following the population code are the number of individuals sampled. Pie charts show the lineage composition of each population at *K* = 2 as inferred using Admixture. Blue and green color represent the West and East lineages, respectively. (b) Comparison of nucleotide diversity (*π*) between the East and West lineages (Wilcoxon–Man–Whitney *U*‐test). (c) The orange bars indicate genetic regions under positive selection region identified by CEGA (*Nλ* < −2.3263). (d) Spearman's correlation between *F*_ST_, *D*_XY_ and average *π*. Orange points are positive selection regions in (c). ***p < 0.001.

Our Admixture analysis based on whole‐genome resequencing data revealed that, under the assumption of *K* = 2, *B. sinensis* populations could be divided into East and West lineages according to their geographic distribution (Figure 1a), although further genetic structure exists within each lineage (Figure S1). This was consistent with the PCA result (Figure S2). The genetic diversity (π) of the 13 populations ranged from 0.0341 (NP) to 0.0708 (LQ), averaging at 0.0636 (Figure S3). After removing admixtures and accounting for sample corrections, we compared π between lineages and found that the West lineage (0.0881) was significantly higher (*p* < 0.001) than the East lineage (0.0671) (Figure 1b). CEGA identified a number of genomic regions under significant positive selection (*Nλ* < −2.3263) (Figure 1c). Although Tajima's *D* values were negative for both the East (−0.6719) and West lineages (−0.5305) (Figure S4), there was an extremely significant difference (*p* < 0.001) between the two lineages. This suggests that the lineages might possess distinct demographic histories. We observed a strong genomic divergence between the two lineages, with respective average *F*_ST_ and *D*_XY_ values of 0.351 and 0.134 (Figure 1d). We then scanned the genes in the positively selected regions, identifying 464 selected genes (Table S2). Functional enrichment analysis of the selected genes determined most to be significantly enriched in pathways related to growth and hypoxia resistance, such as stem cell division (GO:0017145) and response to oxygen levels (GO:0070482) (Tables S3, S4). In addition, we found a significant positive correlation between *F*_ST_ and *D*_XY_ (*R* = 0.67, *p* < 0.001), a significant negative correlation between *F*_ST_ and average *π* (*R* = −0.53, *p* < 0.001), and a significant positive correlation between *D*_XY_ and average *π* (*R* = 0.12, *p* < 0.001) (Figure 1d). These genomic landscapes were largely consistent with the “divergence with gene‐flow” evolutionary scenario proposed by Shang et al. (2023).

**FIGURE 2:**
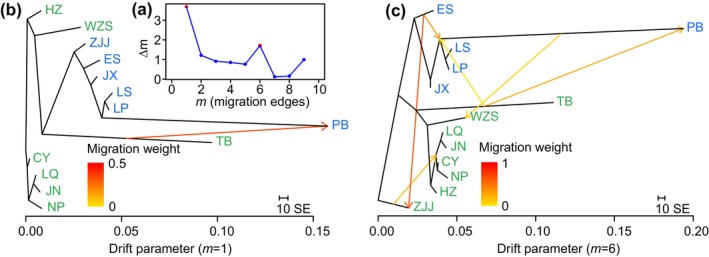
Gene flow inference. (a) Distribution of ∆m inferred by OptM. Gene flow scenarios in Treemix at *m* = 1 (b) and *m* = 6 (c).

To understand the divergence patterns between the two lineages, we inferred probable gene flow scenarios among populations based on the ∆m values. The two most likely gene flow scenarios for Treemix inference (i.e., *m* = 1 and *m* = 6) are depicted (Figure 2, Figure S5). Both scenarios in Treemix included migration events between Eastern and Western populations. This inference was further supported by the ABBA‐BABA test, which detected a total of 333 significant gene flow events across 715 topologies of all populations, including 260 events between the East and West lineages (Table S5).

### 3.2 Demographic History of the East and West Lineages

**FIGURE 3:**
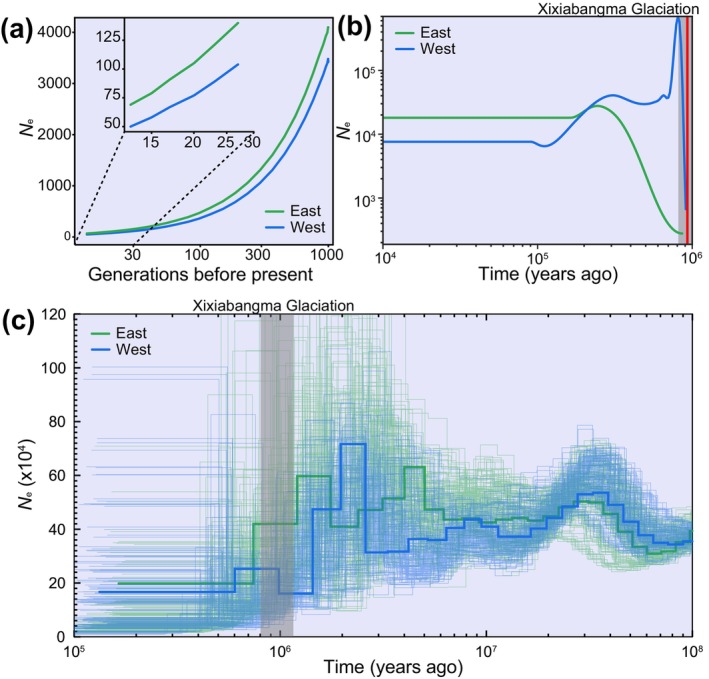
Demographic history of East and West lineages. Historical *N*_e_ changes inferred by SNeP (a), SMC++ (b) and PSMC (c). Gray shading indicates Xixiabangma Glaciation (1.17–0.8 Ma). A thin line in (c) represents one replicate analysis of an individual, and the thick line is the average of all replicates in the lineages.

The SNeP results indicated a consistently slight higher effective population size (*N*_e_) in the East lineage compared to the West lineage over the past 1000 generations, albeit both exhibited a similar declining trend (Figure 3a). The findings of both SMC++ and PSMC analyses further corroborated this, confirming that the East lineage had a higher recent *N*_e_ than the West lineage. However, in ancient times, the West lineage reached a higher peak *N*_e_ than the East lineage (Figure 3b,c). Meanwhile, SMC++ and PSMC unveiled different demographic histories for the two lineages. According to SMC++ analysis, the two lineages diverged approximately 0.9 million years ago (Ma) during the Xixiabangma Glaciation (1.17–0.8 Ma). Following this divergence, the West lineage underwent rapid expansion and contraction, while the East lineage experienced gradual expansion and contraction (Figure 3b). PSMC analysis suggests that both lineages underwent multiple bottlenecks throughout their history, however, their periods of expansion and contraction were not always concurrent, particularly from 6 Ma to Xixiabangma Glaciation (Figure 3c).

### 3.3 Genomic Signatures of Inbreeding and Mutation Load

**FIGURE 4:**
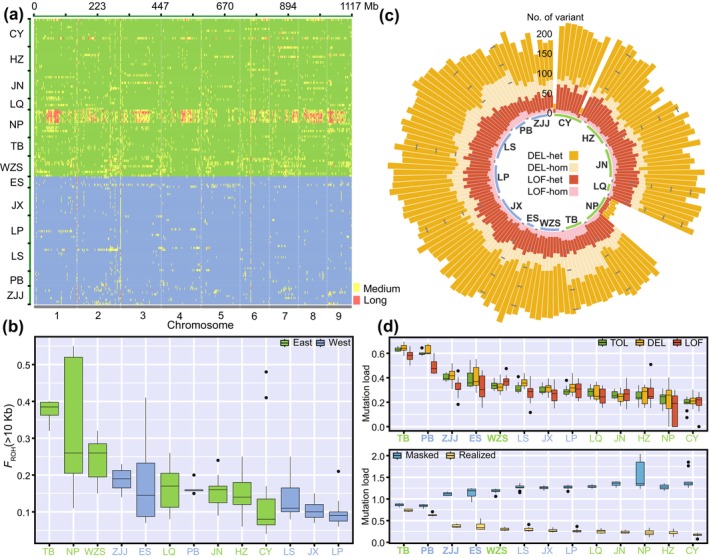
Genomic inbreeding and mutation load assessment of *B. sinensis*. (a) Distribution of medium and long ROHs on the genome of all individual. (b) Average *F*_ROH_ of each population. (c) Number of detected DEL and LOF mutations on all individual. (d) Mutation loads calculated based on TOL, DEL and LOF mutations, and masked loads and realized loads calculated based on deleterious heterozygous and homozygous mutations. The bolded fonts in (d) are the five‐edge populations (see Figure 1a).

A total of 1,506,451 short ROHs, 9695 medium ROHs, and 874 long ROHs were identified across 154 *B. sinensis* individuals (Figure 4a, Table S6). Notably, a significant accumulation of long ROHs was found in several individuals from the NP population. Several ROH hotspots were also detected within the genome, specifically in regions close to 112 Kb on chromosome 2 and 38 Mb on chromosome 6, suggesting that numerous individuals shared specific ROH regions (Figure 4a, Table S7). The mean *F*_ROH_ coefficients show that the TB population from the East exhibit the highest levels of individual inbreeding, as most short ROHs were also contribute to *F*_ROH_, while the LP population from the West had the lowest (Figure 4b). When comparing individual‐based *F*_IS_ with *F*_ROH_, it was observed that the former tends to overestimate the level of inbreeding (Figure S6). Nonetheless, both coefficients displayed a similar trend and had an extremely significant positive correlation (*R* = 0.57, *p* < 0.001) (Figure S7).

Four types of mutation were identified based on their level of deleteriousness. Most prevalent among these were SYN and TOL mutations (Table S7). In contrast, DEL and LOF mutations were found less abundant, with individual counts ranging from 23 to 268 (Figure 4c). Mutation loads, calculated considering TOL, DEL, and LOF mutations, indicated that all five edge populations (i.e., TB, PB, WZS, ZJJ, and ES) had surpassed those of the center (Figure 4d, Table S7). A similar trend was observed with realized loads, whereas the masked load presented an inverse relationship with the realized load.

### 3.4 Genomic Offset to Future Climate Change

**FIGURE 5:**
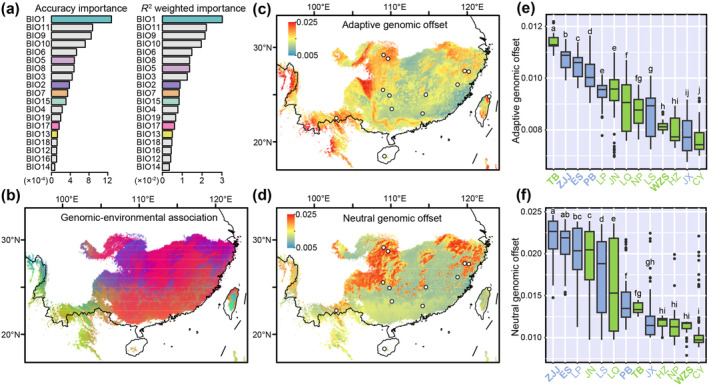
Genomic offset to future climate change in *B. sinensis*. (a) Accuracy importance and *R* ^2^ weighted importance ranking of 19 climate variables in GF models. The colors are the seven representative variables. (b) Genomic‐environmental association across the distribution area. Colors represent the PCA‐summarized gradients in genomic turnover. The first three principal components are represented by red, green, and blue RGB colors, respectively. Landscape of genomic offsets across the distribution range in 2081–2100 estimated using adaptive SNPs (c) and neutral SNPs (d). Mean adaptive (e) and neutral (f) genomic offsets of each population. Different letters indicate significant differences (*p* < 0.05) after Duncan's multiple comparison test. The bolded fonts in (e, f) are the five edge populations (see Figure 1a).

We employed GF analysis to determine the relative importance of 19 bioclimatic variables in explaining the distribution of genetic diversity in *B. sinensis* (Figure 5a). The genomic‐environmental association in our GF model were mapped across the distribution area, with the first three principal components accounting for a cumulative explanation rate of 96.6% (Figure 5b, Figure S8). To reduce redundancy, seven representative variables were retained for estimating genomic offsets. These variables include annual mean temperature (BIO1), mean diurnal range (BIO2), max temperature of warmest month (BIO5), temperature annual range (BIO7), precipitation of wettest month (BIO13), precipitation seasonality (BIO15), and precipitation of driest quarter (BIO17) (Figures S9–S12). A total of 2334 adaptive SNPs were identified by the RDA model (Figures S13, S14, Table S8). Correspondingly, we also randomly selected 2334 neutral SNPs. Across the distribution of *B. sinensis*, the average adaptive genomic offset was 0.009, while the average neutral genomic offset was 0.014 (Figure 5c,d, Tables S9, S10). Despite the adaptive genomic offset being marginally less than the neutral genomic offset, the edge populations of the species appear to show a high degree of maladaptation to anticipated climate change in both scenarios. As a result, we further quantified the genomic offsets of each population and found that the four edge populations (TB, ZJJ, ES, and PB) had significantly higher adaptive genomic offsets than the other populations. Notably, two edge populations (ZJJ and ES) also ranked highest in neutral genetic offsets (Figure 5e,f).

**FIGURE 6:**
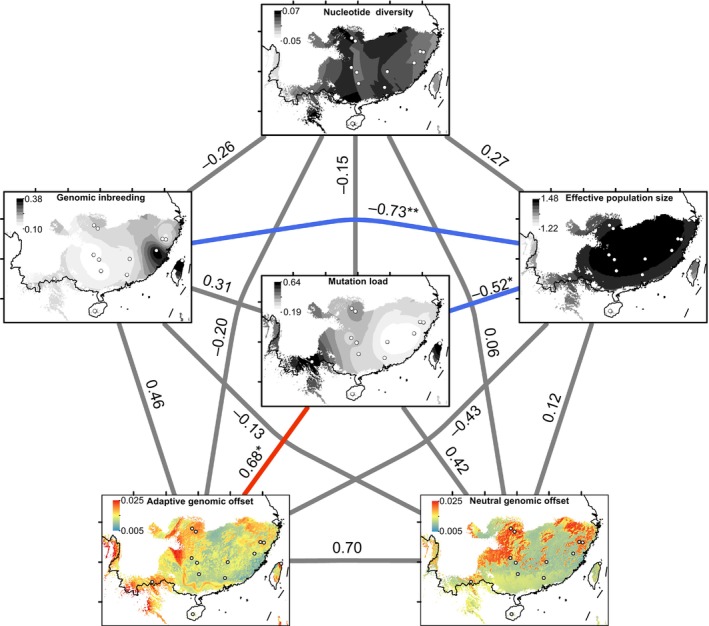
Relationships between genomic offsets, mutation load, genomic inbreeding, nucleotide diversity, and effective population size. Adaptive and neutral genomic offsets are from Figure 5c,d. Mutation load is calculated using the DEL and LOF variants together. *F*_ROH_ is the genomic inbreeding coefficient. π is the nucleotide diversity. *N*_e_ is the effective population size of the most recent generation of each population inferred using SNeP, and the values are logarithmically converted. Mutation load, genomic inbreeding, nucleotide diversity, and effective population size are visualized using the Kriging space interpolation method. Red and blue connecting lines indicate significantly positive and negative Spearman's correlations, respectively. *p < 0.05; **p < 0.01.

Finally, we conducted correlation analyses between adaptive genomic offset, neutral genetic offset, mutation load, π, *F*_ROH_, and *N*_e_ by calculating their Spearman's correlations. The results revealed a significant positive correlation (*R* = 0.68, *p* < 0.05) between adaptive genomic offset and mutation load, a significant negative correlation between *N*_e_ and mutation load (*R* = −0.52, *p* < 0.05), and a significant negative correlation between *F*_ROH_ and *N*_e_ (*R* = −0.73, *p* < 0.01). No statistically significant correlation (*p* > 0.05) was found between other indicators (Figure 6).

## 4 Discussion

### 4.1 High Differentiation and Different Demographic Histories of East and West Lineages

Our genome‐wide population structure analysis revealed that *B. sinensis* diverged into East and West lineages, following their geographic distribution. This finding is also consistent with a recent study using chloroplast sequences (Shang et al. 2022), and their estimated divergence time (0.6 Ma, by BEAST) is close to our estimation of 0.9 Ma (by SMC++). The genomic differentiation between the East and West lineages of *B. sinensis* was substantial (mean *F*_ST_ = 0.351) compare with other tree species in subtropical forests. For example, the *F*_ST_ between the South and North lineages of *D. sinensis*, another widely distributed relict species, was recently found to be only 0.04 (Feng et al. 2024). The *F*_ST_ for the East and West lineages of *Quercus acutissima* was even smaller (*F*_ST_ = 0.024; Yuan et al. 2023). It appears that the driving factors of lineage differentiation across various species in East Asia may differ (Kong et al. 2022; Yin et al. 2021; Wang et al. 2023). For example, the topographic differences between China's mountainous West and low‐lying East, combined with climatic variations amplified by the monsoon's enhancement since the Miocene, are believed to have significantly influenced the diversification of plant lineage in East Asia (Kong et al. 2022). Additionally, we found that *B. sinensis* experienced multiple historical bottlenecks, which likely contributed to the high divergence between lineages. Although previous studies have documented recurrent bottlenecks in this species (Liu et al. 2022; Zhang et al. 2022), none have revealed differences in the demographic histories between lineages. Our analysis reveals that East and West lineages underwent multiple asynchronous bottlenecks, becoming substantially separated during the most recent bottleneck in the Xixiabangma Glaciation (1.17–0.8 Ma). Based on the genomic landscapes of *F*_ST_, *D*_XY_, and *π*, we hypothesize that the genomic divergence between the East and West lineages fits a scenario of divergence with gene flow (Shang et al. 2023). This hypothesis is also supported by the Treemix and Dsuite inferences. These results indicate that the inferred gene flow between East and West populations might have occurred either before or early during the lineage differentiation, when *N*_e_ was significantly higher than it is currently.

We identified several genes associated with hypoxia response under positive selection (Table S2). These genes are shown to play important roles in facilitating plant adaptation to hypoxic conditions (Ma et al. 2022). In natural environments, plants may experience hypoxic or anoxic stress when soil saturation occurs after heavy precipitation (Habibi et al. 2023). As wild *B. sinensis* populations usually occur in valleys and streamside slopes (Gong et al. 2015), these moist habitats could subject plant roots to hypoxia during rainy seasons. Therefore, genes associated with hypoxia response may have been positively selected to facilitate adaptation to moist habitats. A recent study by Ma et al. (2022) found that mangrove plants of the *Acanthus* genus exhibit similar positive selection patterns on hypoxia‐responsive genes during their migration from terrestrial to moist intertidal habitats. This suggests the possibility of conserved molecular mechanisms for plant adaptation to moist habitats.

### 4.2 High Mutation Load and Genomic Offset in Edge Populations

Mutation load offers a crucial method for assessing susceptibility to genetic threats in small populations (Robinson et al. 2023). Populations at the geographical periphery of a species' range, known as edge populations, usually face different pressures in comparison to populations in closer proximity to the center of the species' range. These edge populations tend to be smaller, more isolated, and often exist in less favorable environmental conditions (Hampe and Petit 2005). Such circumstances can lead to increased genetic drift and inbreeding, both potentially significantly amplifying their mutation load (Angert, Bontrager, and Ågren 2020). While recent studies suggest that strongly deleterious mutations can be purged from a population over time (Xue et al. 2015; Robinson et al. 2018; Grossen et al. 2020; Mathur and DeWoody 2021; Tao et al. 2024), the effectiveness of genetic purging in natural populations remains a subject of debate. Our spatial interpolation simulations revealed a gradient of decreasing *N*_e_ as distance from the center increased (Figure 6). Consequently, reduced *N*_e_ in edge populations renders purifying selection ineffective against deleterious mutations. Furthermore, strong drift at the edge may override the effect of selection, resulting in loss of genetic diversity and accumulation of deleterious mutations (Pironon et al. 2017; Koski et al. 2019). Mutation load is also predicted to increase during range expansions (known as expansion load), which can significantly impair further expansion and will persist in the area of past expansion for thousands of generations (Peischl et al. 2013; Peischl, Kirkpatrick, and Excoffier 2015). Consistent with this expectation, a number of empirical studies on the geographic distribution pattern of mutation load have demonstrated that edge populations tend to accumulate more mutation load (Zhang et al. 2016; Willi et al. 2018; Willi 2019; Jiang et al. 2024). A similar geographic distribution pattern of mutation load was observed in our case study of the relict tree species *B. sinensis* (Figure 4d). High mutation load on edge can reduce the rate of range expansion, which helps maintain species distribution limit (Angert, Bontrager, and Ågren 2020). This might explain the scarcity of this relict species outside of China. A previous phylogeographic study suggested that the East Yunnan‐Guizhou Plateau and Nanling Mountains may have served as glacial refugia for *B. sinensis* (Hu et al. 2017). Our results reinforced the previous findings and speculated that outward expansion from post‐glacial refugia might have played a key role in shaping the current geographic distribution patterns of mutation load and genetic diversity.

Climate change poses a substantial challenge to the survival and distribution of relict species. Evaluating genomic offset, which measure the discrepancy between a population's existing genetic diversity and what would be expected under optimal conditions, can help in predicting the possible fate of natural populations under future climate change scenarios (Fitzpatrick and Keller 2015). Our result of GF modeling has shown that edge populations generally have high genomic offsets (Figure 5). This indicates a need for substantial changes in adaptive allele frequencies to maintain the observed genetic–environmental association in these edge populations. These findings are consistent with previous studies showing that marginal populations of forest trees, such as *B. sinensis*, exhibit high genomic vulnerability to the rapidly climate changes (Fréjaville et al. 2020; Gougherty, Keller, and Fitzpatrick 2021; Ingvarsson and Bernhardsson 2020; Jia et al. 2020; Wang et al. 2023; Yuan et al. 2023). Given the long generation times of *B. sinensis*, a low rate of allele frequency turnover may hinder its ability to adapt to the rapidly changing climate. It is worth noting that our GF predictions of genomic vulnerability are based on a statistical simplification of genotype‐environment associations and do not account for other factors such as phenotypic plasticity, migration or future adaptation that could buffer populations against climate change. As such, the results should be interpreted with caution. Given the limitation of the genomic offset approach, Rellstab, Dauphin, and Exposito‐Alonso (2021) suggest using it in combination with other approaches (e.g., experiments, ecological niche modeling, and genetic diversity assessments) to inform conservation actions. For example, Borrell et al. (2020) found that dwarf birch populations (*Betula nana*) with high risk of nonadaptedness (e.g., high degree of genomic offset) were particularly small, isolated, and at the margins of the species' distribution. As expected, our study found a positive correlation (*R* = 0.68, *p* < 0.05; Figure 6) between adaptive genomic offset and mutation load in *B. sinensis*, indicating that less‐fit populations (as measured by mutation load) are more genetically vulnerable to future climate change. In agreement with the previous findings of negative associations between genomic offset and population size (Bay et al. 2018; Ruegg et al. 2018), we observed a negative but not significant correlation (*R* = −0.43, *p* = 0.07; Figure 6) between adaptive genomic offset and *N*_e_. These results suggest that, due to the low *N*_e_ in the edge populations, increased genetic drift may have led to greater allele frequency turnover at the climatic margins of the species. Recent studies have shown that randomly sampled loci in the genome are as good as, or even better than, adaptive loci at predicting climate maladaptation (Fitzpatrick et al. 2021; Lind et al. 2024). However, despite a high correlation between neutral and adaptive genomic offsets in *B. sinensis*, this correlation is not statistically significant (*R* = 0.70, *p* = 0.17; Figure 6). Therefore, our study suggests that it remains important to differentiate between adaptive and neutral variants when predicting genomic vulnerability in response to future climate changes. Collectively, our results suggest that the edge populations of *B. sinensis* face a higher risk of local extinction due to climate change and should be considered a priority for conservation efforts.

## 5 Conclusions

In this study, we investigated lineage divergence, mutation load and genomic offset in the relict tree *B. sinensis* in subtropical forests. We delineated two highly divergent lineages, each manifesting different demographic histories. We observed that several genes associated with growth and hypoxia response underwent positive selection during lineage differentiation, highlighting their potential role in the species' survival and adaptation under differing ecological conditions. Our investigation into genomic vulnerability revealed distinct geographic distribution patterns of mutation load accumulation and genomic offset. These findings serve as indicators of a population's genetic robustness and its capacity to respond to environmental changes respectively. Notably, we detected higher levels of overall and realized mutation load in edge populations, indicating a heavier burden of harmful genetic variants. Furthermore, these populations generally exhibited high genomic offsets in relation to future climate change, implying a mismatch between the population's genetic adaptations and anticipated environmental shifts. These findings imply that edge populations of *B. sinensis* are particularly vulnerable, posing a higher risk for local extinction under future climate change. This study provides critical insights for the conservation planning of *B. sinensis*, emphasizing the importance of incorporating genetic health and adaptability into conservation strategies. Further studies should seek to explore proactive measures, such as the facilitation of gene flow or assisted migration, to enhance the resilience of these vulnerable populations against the impending climate change impacts.

## Conflicts of Interest

The authors declare no conflicts of interest.

## Supporting information


**Figure S1.** Population structure detected by Admixture with different *K* values.
**Figure S2.** Results of principal component analysis (PCA).
**Figure S3.** Average nucleotide diversity (*π*) of each population.
**Figure S4.** Comparison of Tajima’ *D* values between the East and West lineages.
**Figure S5.** Standard errors (SE) for *m* = 1 and *m* = 6 in Treemix analysis.
**Figure S6.** Individual‐based inbreeding coefficients (*F*_IS_) in each population.
**Figure S7.** Spearman’s correlation between *F*_ROH_ and inbreeding coefficients (*F*_IS_).
**Figure S8.** PCA of the genomic variation predicted by the GF model across the species range.
**Figure S9.** Current and future (SSP585) distributions of seven representative climate variables.
**Figure S10.** Spearman’s correlation between seven representative climate variables.
**Figure S11.** Cumulative importance of seven representative climate variables in gradient forest models.
**Figure S12.** Split density of seven representative climate variables in gradient forest models.
**Figure S13.** Frequency of SNP distribution and explanatory rate in the first three RDAs.
**Figure S14.** Adaptive SNPs identified in RDA models.

**Table S1.** Information for each SNP dataset.
**Table S2.** Information of selected genes.
**Table S3.** GO enrichment analysis of selected genes.
**Table S4.** KEGG enrichment analysis of selected genes.
**Table S5.** Estimates of introgression across different populations based on ABBA‐BABA test.
**Table S6.** Information on medium and long ROHs detected by Plink.
**Table S7.** Number of mutations and mutation load in each individual.
**Table S8.** Adaptive SNPs identified using the RDA model.
**Table S9.** Genomic offsets under four future climate scenarios using 2334 adaptive SNPs.
**Table S10.** Genomic offsets under four future climate scenarios using 2334 neutral SNPs.

## Data Availability

The data that support the findings of this study are openly available at the National Center for Biotechnology Information Sequence Read Archive (https://www.ncbi.nlm.nih.gov/bioproject/PRJNA721741/).
