# Supplementary material for: Lineage Differentiation and Genomic Vulnerability in a Relict Tree From Subtropical Forests
Source: Evol Appl. 2024 Nov 1;17(11):e70033. doi: 10.1111/eva.70033 (PMC11530410; doi:10.1111/eva.70033)
Supplement: Supplementary file 1 — Figure S1. Population structure detected by Admixture with different K values. Figure S2. Results of principal component analysis (PCA). Figure S3. Average nucleotide diversity (π) of each population. Figure S4. Comparison of Tajima’ D values between the East and West lineages. Figure S5. Standard errors (SE) for m = 1 and m = 6 in Treemix analysis. Figure S6. Individual‐based inbreeding coefficients (F IS) in each population. Figure S7. Spearman’s correlation between F ROH and inbreeding coefficients (F IS). Figure S8. PCA of the genomic variation predicted by the GF model across the species range. Figure S9. Current and future (SSP585) distributions of seven representative climate variables. Figure S10. Spearman’s correlation between seven representative climate variables. Figure S11. Cumulative importance of seven representative climate variables in gradient forest models. Figure S12. Split density of seven representative climate variables in gradient forest models. Figure S13. Frequency of SNP distribution and explanatory rate in the first three RDAs. Figure S14. Adaptive SNPs identified in RDA models. [file EVA-17-e70033-s002.docx]

**Supplementary Figures**


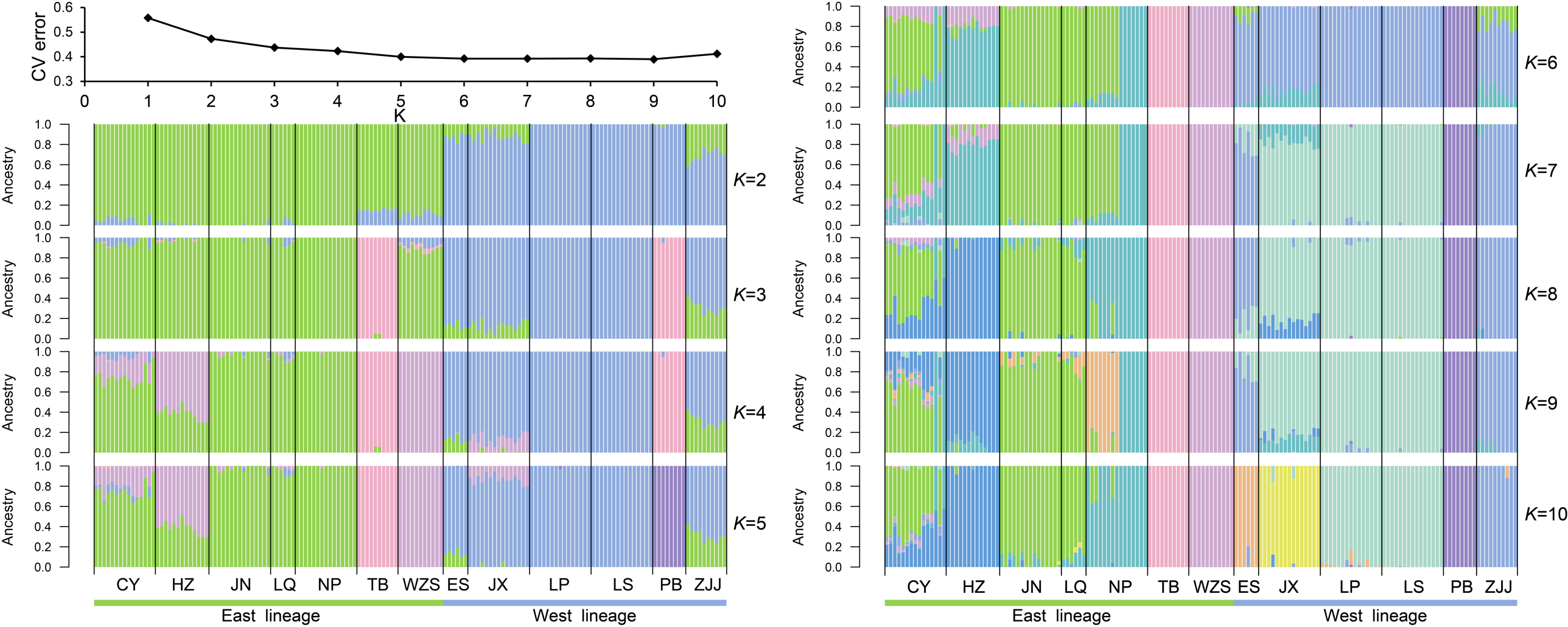


## Figure S1. Population structure detected by Admixture with different *K* values.


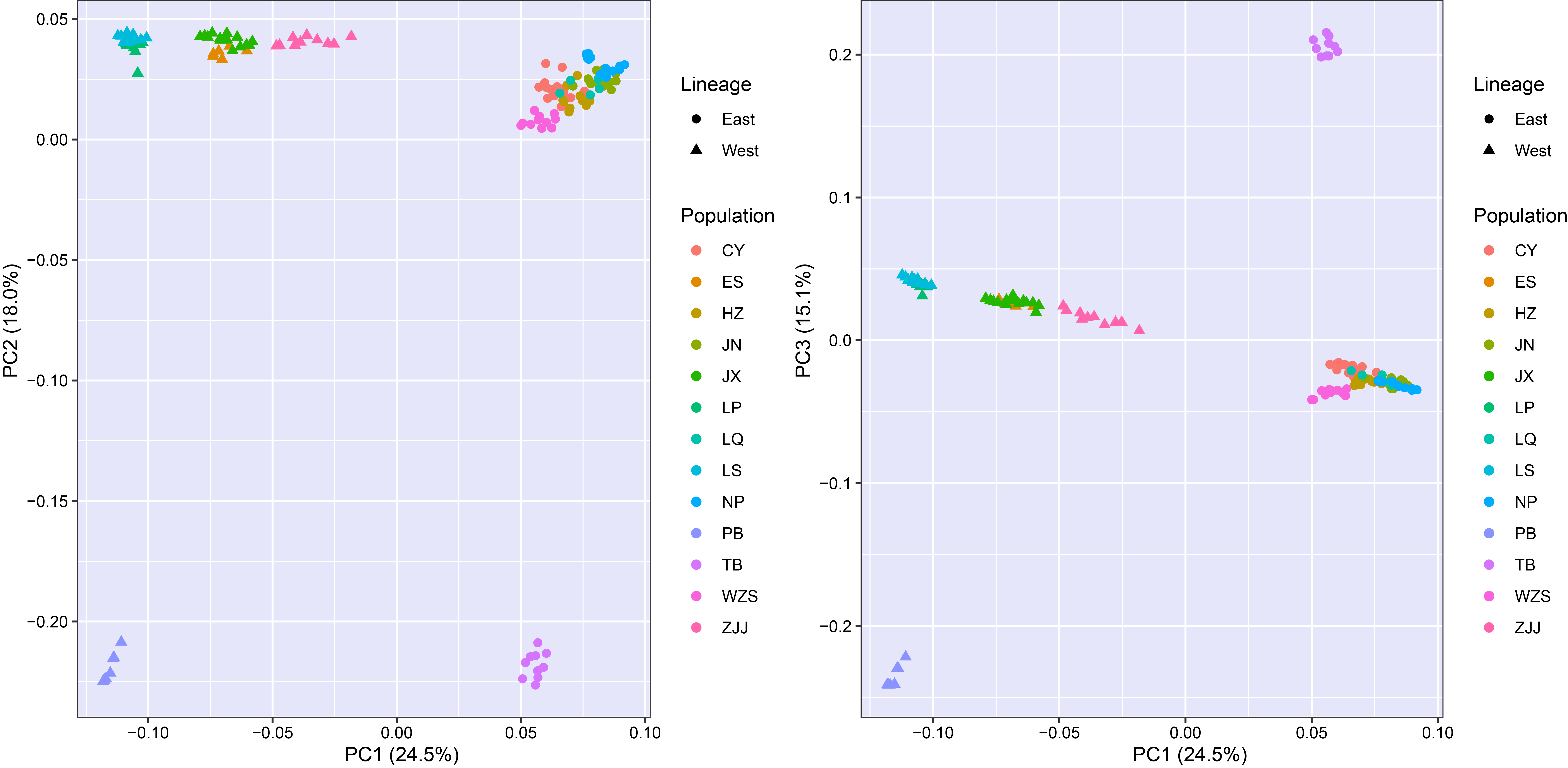


## Figure S2. Results of principal component analysis (PCA).


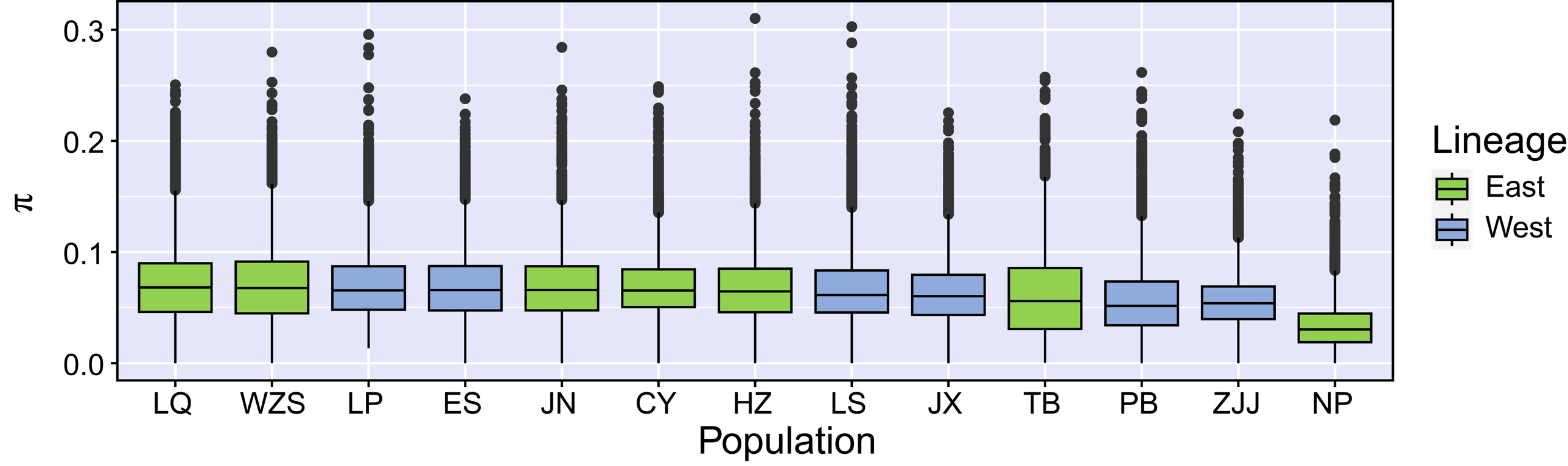


## Figure S3. Average nucleotide diversity (π) of each population.


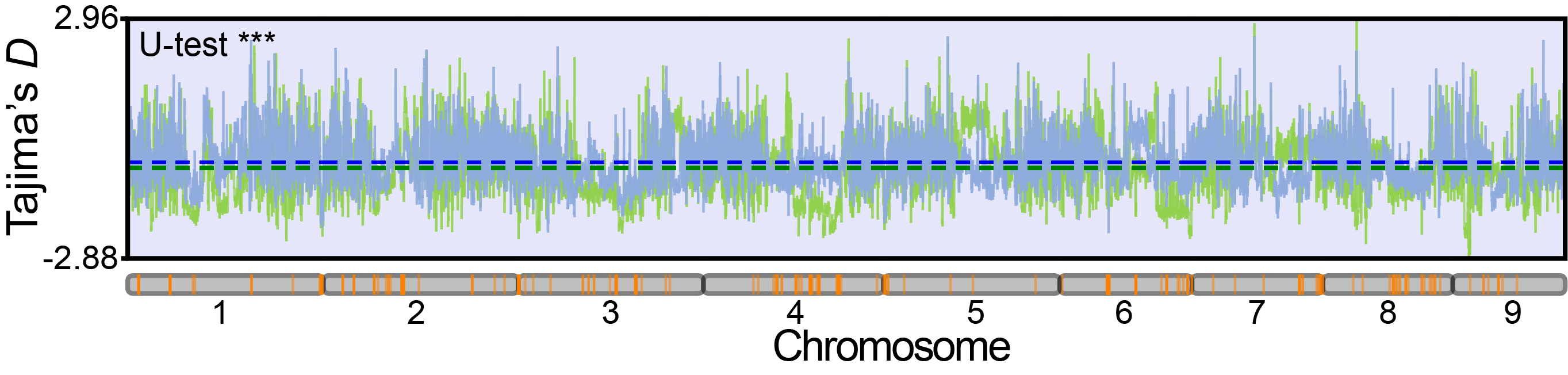


## Figure S4. Comparison of Tajima's *D* values between the East and West lineages.


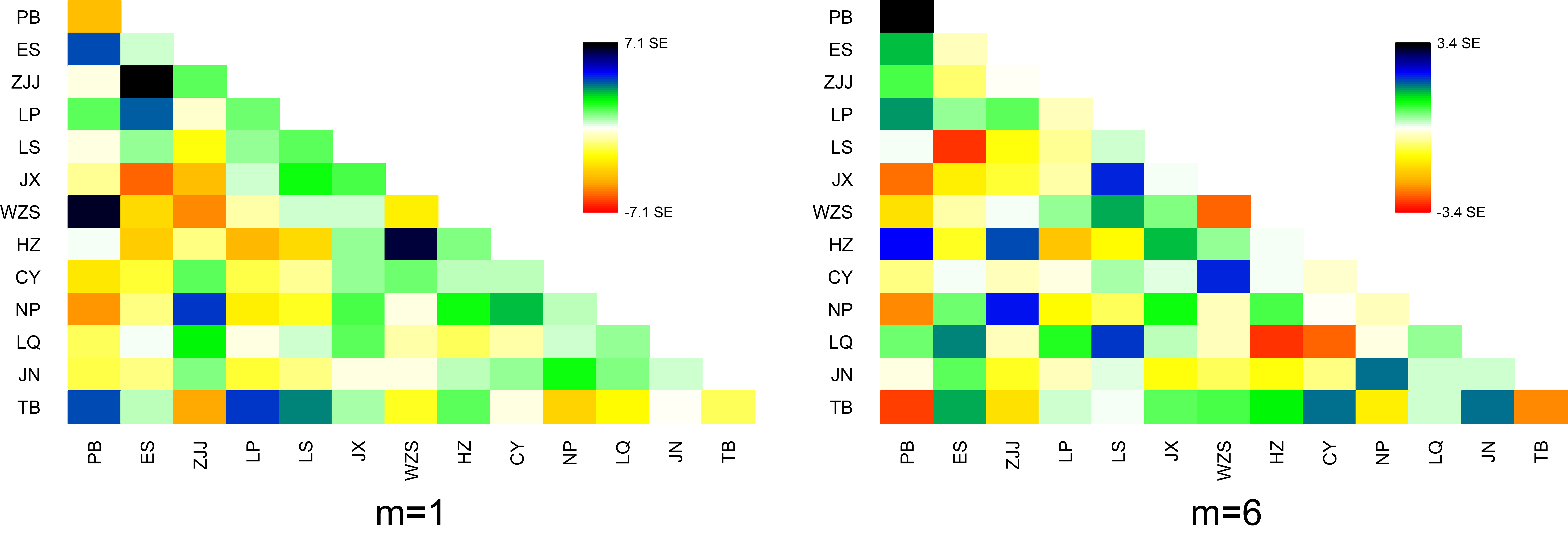


## Figure S5. Standard errors (SE) for m = 1 and m = 6 in Treemix analysis.


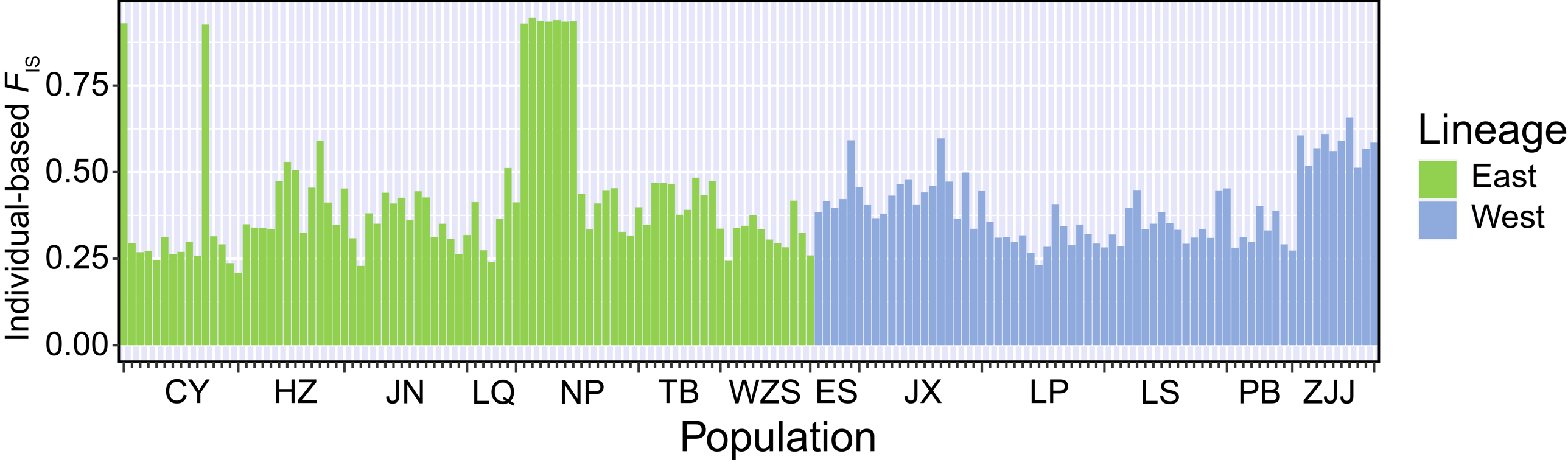


## Figure S6. Individual-based inbreeding coefficients (*F*_IS_) in each population.


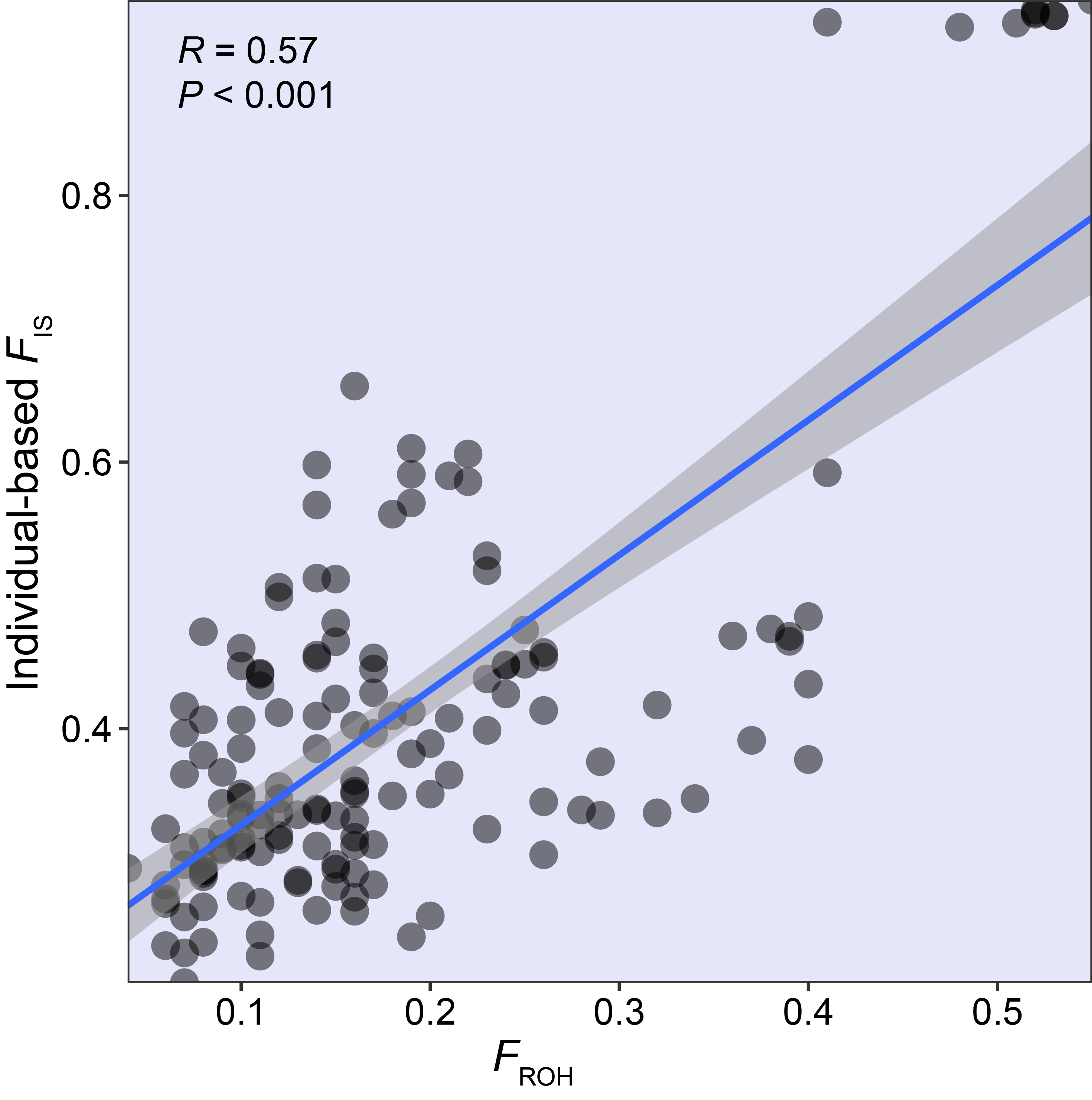


## Figure S7. Spearman's correlation between *F*_ROH_ and inbreeding coefficients (*F*_IS_).


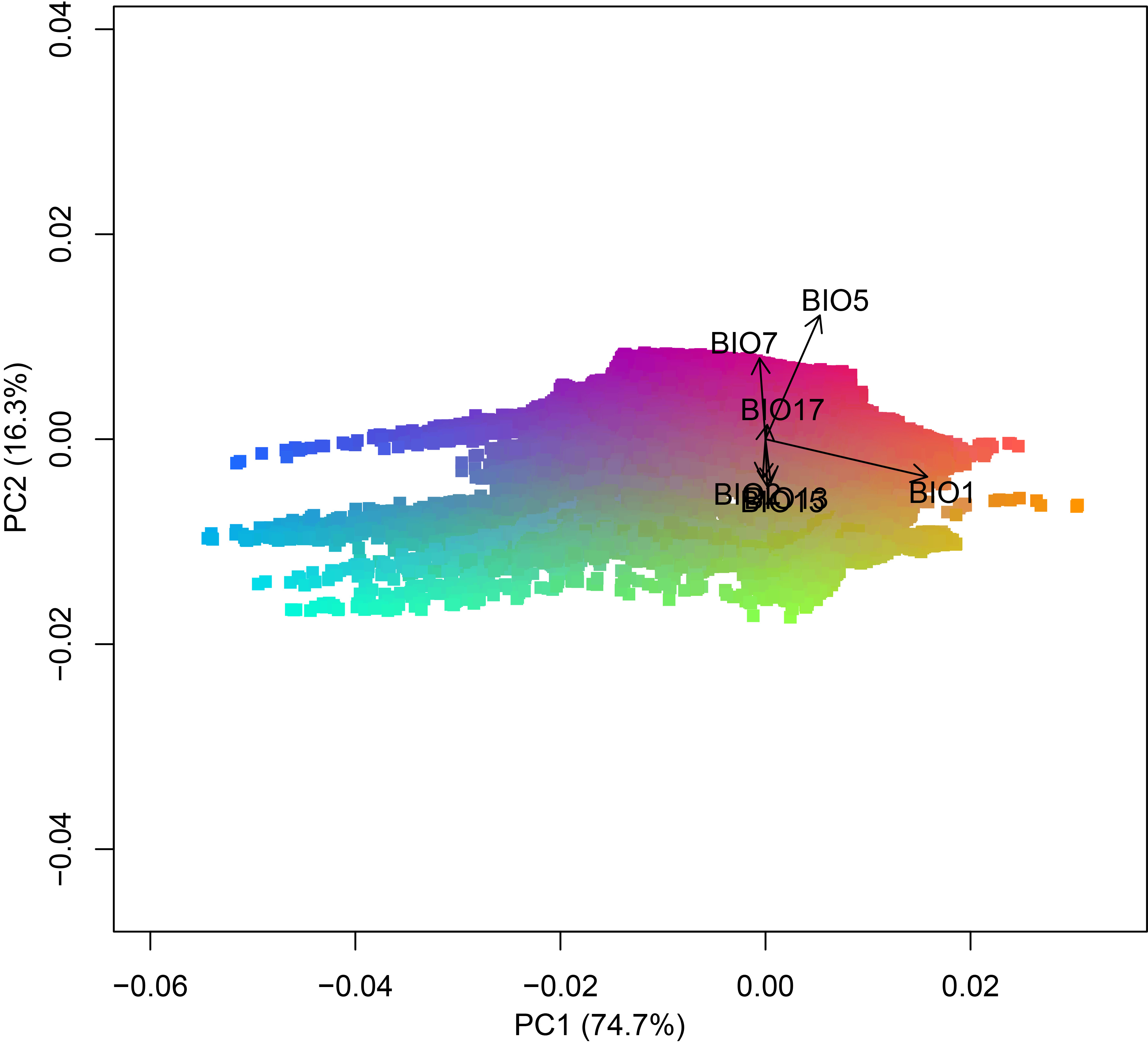


## Figure S8. PCA of the genomic variation predicted by the GF model across the species range. The loadings are the seven representative climate variables.





## Figure S9. Current and future (SSP585) distributions of seven representative climate variables. BIO1, annual mean temperature. BIO2, mean diurnal range. BIO5, max temperature of warmest month. BIO7, temperature annual range. BIO13, precipitation of wettest month. BIO15, precipitation seasonality. BIO17, precipitation of driest quarter. The same below.


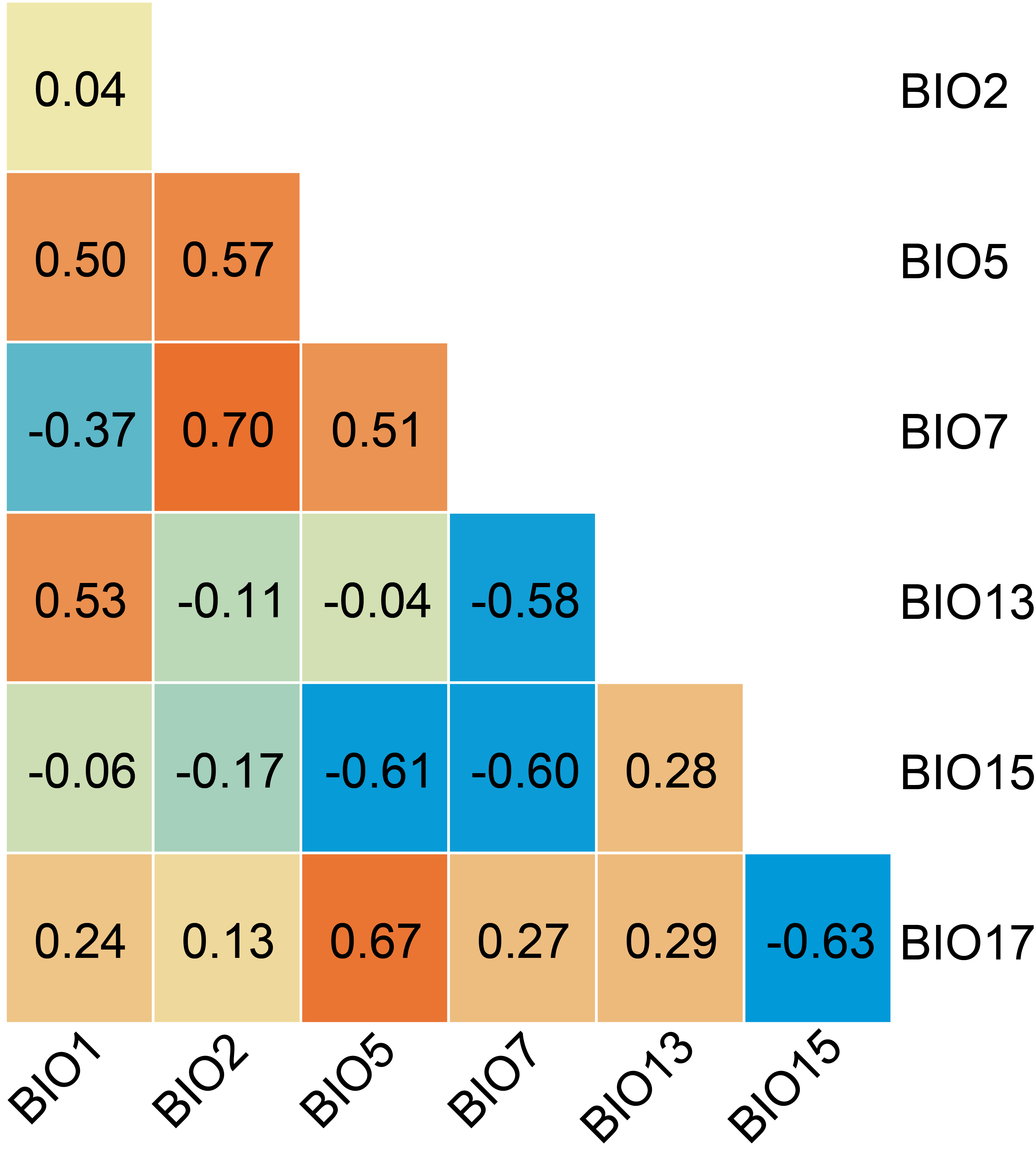


## Figure S10. Spearman's correlation between seven representative climate variables.


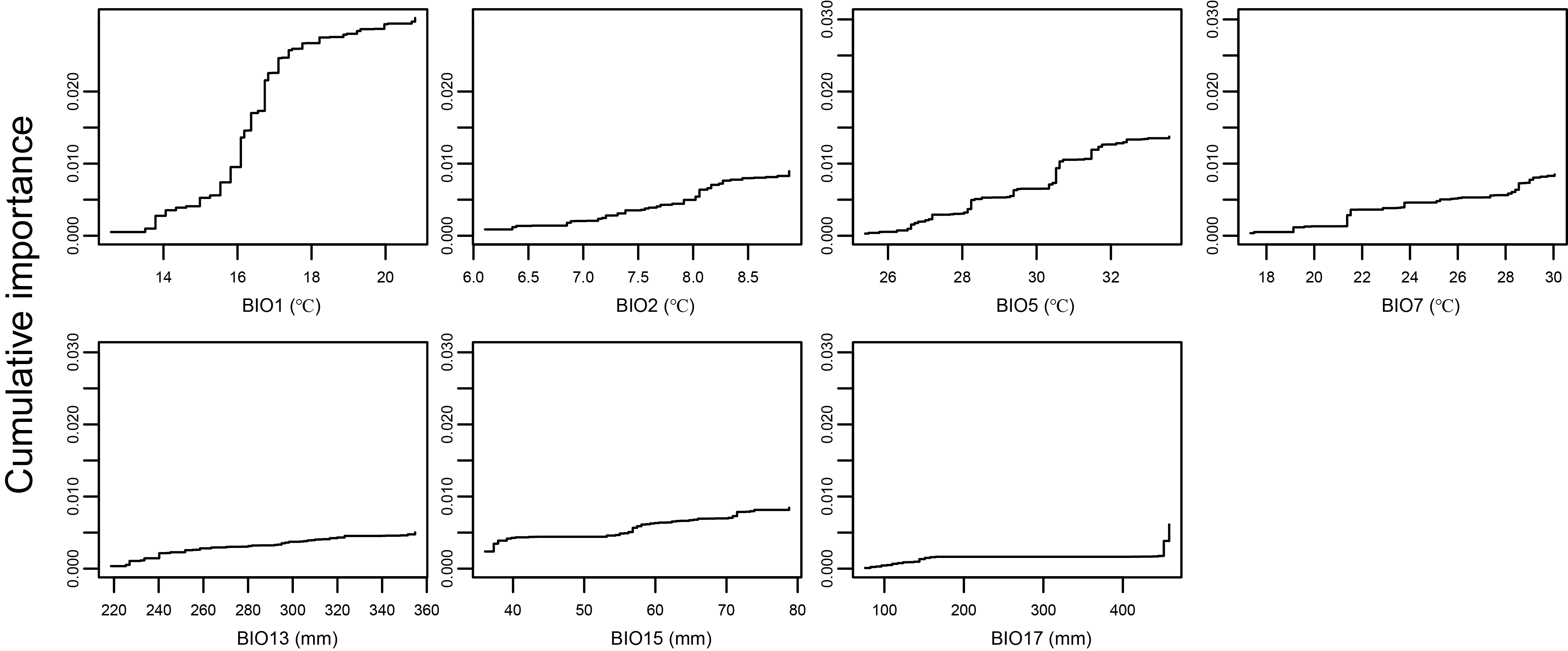


## Figure S11. Cumulative importance of seven representative climate variables in gradient forest models.


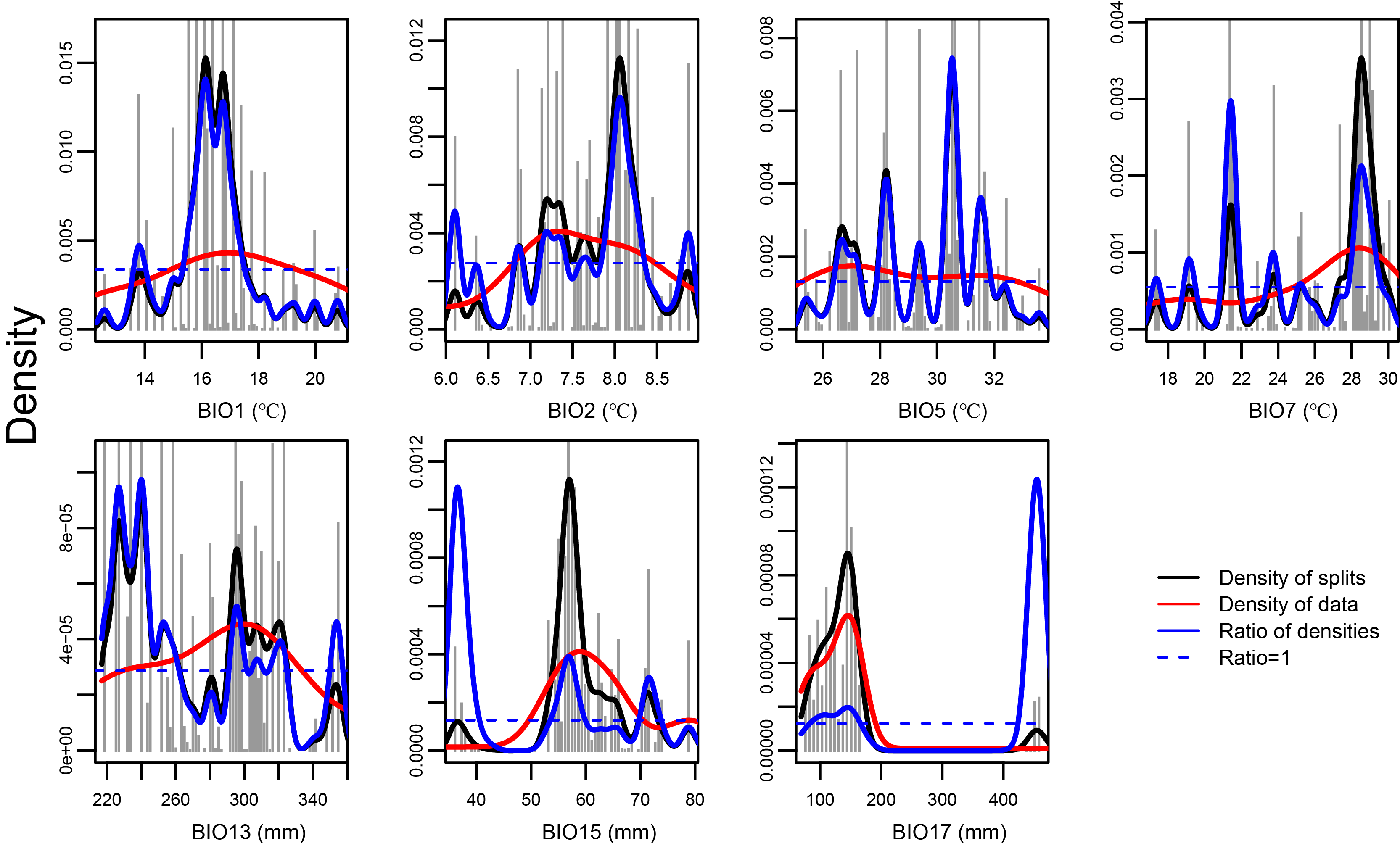


## Figure S12. Split density of seven representative climate variables in gradient forest models.


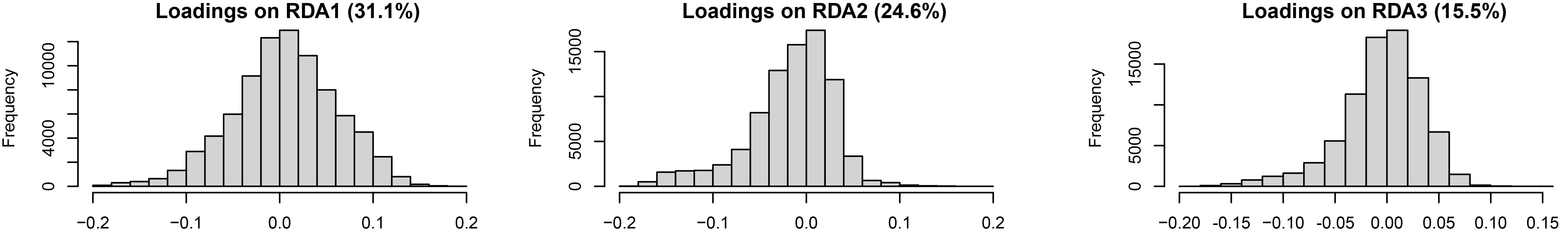


## Figure S13. Frequency of SNP distribution and explanatory rate in the first three RDAs.


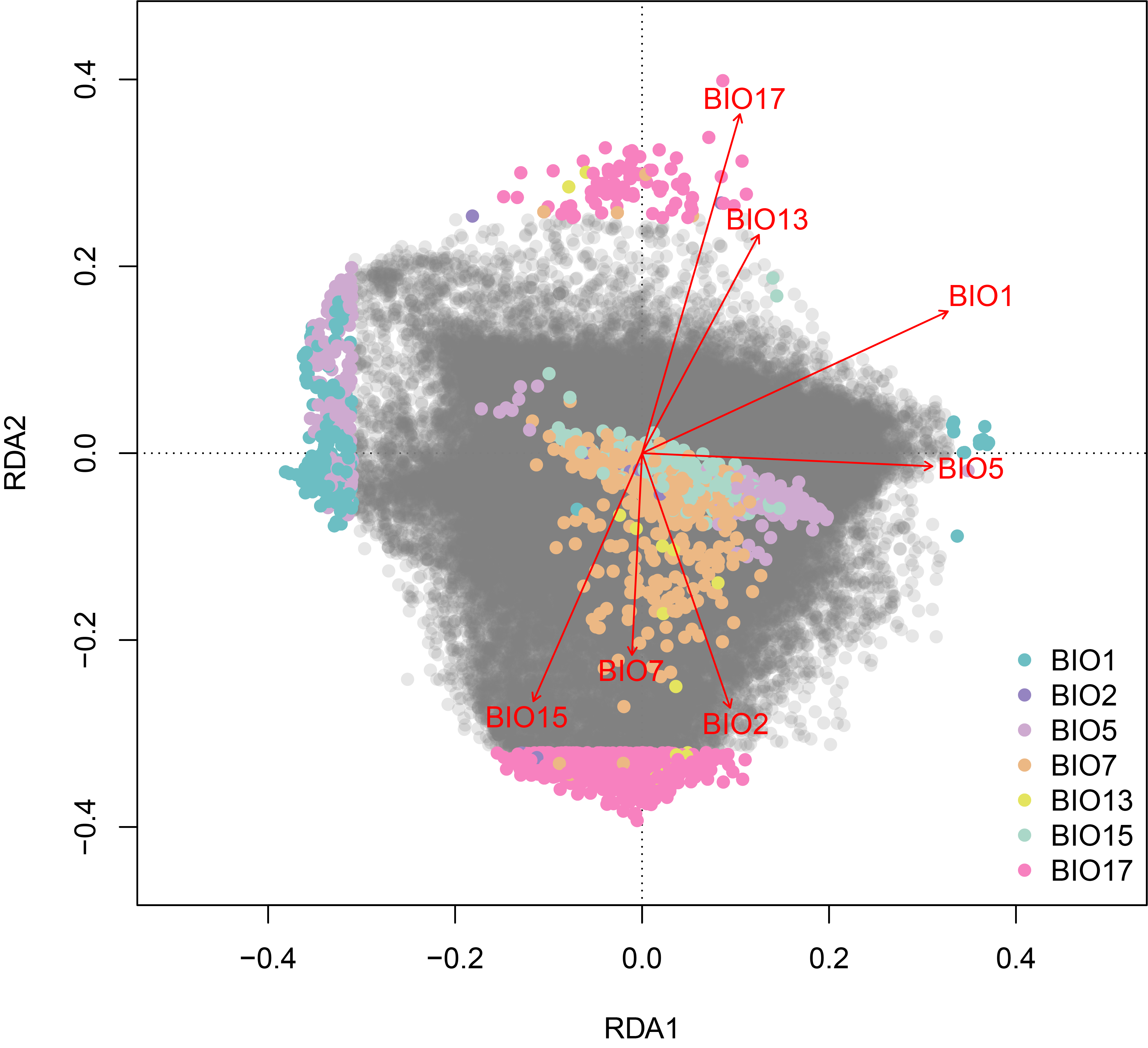


## Figure S14. Adaptive SNPs identified in RDA models. Colored are adaptive SNPs, and grey are non-candidate SNPs.
